# Tetrabutylammonium Bromide (TBABr)-Based Deep Eutectic Solvents (DESs) and Their Physical Properties 

**DOI:** 10.3390/molecules19068011

**Published:** 2014-06-13

**Authors:** Rizana Yusof, Emilia Abdulmalek, Kamaliah Sirat, Mohd Basyaruddin Abdul Rahman

**Affiliations:** 1 Department of Chemistry, Faculty of Science, Universiti Putra Malaysia, 43400 UPM Serdang, Selangor, Malaysia; E-Mails: rizanayusof@gmail.com (R.Y.); emilia@upm.edu.my (E.A.); kamaliah@upm.edu.my (K.S.); 2 Department of Chemistry, Faculty of Applied Sciences, Universiti Teknologi MARA (UiTM), 40450 Shah Alam, Selangor, Malaysia

**Keywords:** deep eutectic solvent, tetrabutylammonium bromide, density, viscosity, ionic conductivity

## Abstract

Density, viscosity and ionic conductivity data sets of deep eutectic solvents (DESs) formed by tetrabutylammonium bromide (TBABr) paired with ethlyene glycol, 1,3-propanediol, 1,5-pentanediol and glycerol hydrogen bond donors (HBDs) are reported. The properties of DES were measured at temperatures between 303 K and 333 K for HBD percentages of 66.7% to 90%. The effects of HBDs under different temperature and percentages are systematically analyzed. As expected, the measured density and viscosity of the studied DESs decreased with an increase in temperature, while ionic conductivity increases with temperature. In general, DESs made of TBABr and glycerol showed the highest density and viscosity and the lowest ionic conductivity when compared to other DESs. The presence of an extra hydroxyl group on glycerol in a DES affected the properties of the DES.

## 1. Introduction

Over the past few decades, ionic liquids (ILs) have been a research topic of interest due to their unique physical properties: low melting point [1,2], low volatility [2,3], high thermal stability [2,3,4], high polarity [3], inflammability [3,4], and wide range of solubility [5,6]. However, there have problems related to high cost, purification and toxicity [1,4,7,8]. These problems have been largely rectified with the emergence of deep eutectic solvents (DESs), an advanced generation of ILs which are biodegradable, cheap and easy to prepare [9,10]. DES are structurally different from ILs, as DES have both ionic and non ionic species and are connected by a hydrogen bonding network. Nevertheless, DES can replace ILs in many applications due to their shared characteristics [9].

Abbott and his group made an excellent discovery by simply mixing choline chloride, C_5_H_14_ClNO (ChCl) and urea, (NH_2_)_2_CO, which produced a DES with a melting point of 285 K [11]. The low melting point is a result of mixing an organic salt and a hydrogen bond donor (HBD) such as an amine, alcohol, amide or carboxylic acid [11,12]. Until now, most DES studies have been primarily focused on the economical and biodegradable substituted quaternary ammonium salts of ChCl [10,13,14,15,16]. ChCl or 2-hydroxyethyltrimethylammonium are produced annually on the Mtonne (million metric ton) scale as a main source of vitamin B4 for chicken feed. It is also known as an essential micronutrient needed for a normal body function and to promote health [17]. Meanwhile, a quaternary ammonium salt, tetrabutylammonium bromide (TBABr) has emerged as a popular phase-transfer catalyst in various organic transformations [18]. The salt has been found to be a valuable source of bromide [19] and is sometimes used as an IL [20]. Not only it is cheap, but TBABr is also environmentally friendly, has greater selectivity, is operationally simple, non-corrosive and easily recyclable [18].

Previously, there has been a little research into the physical properties such as density, viscosity, surface tension, refractive index and pH of synthesized DESs, such as ChCl [13,14], fructose [21] and glucose-based DES [22]. The known properties of phosphonium-based DESs include the melting point, density, viscosity, pH, conductivity and dissolved oxygen content reported by Kareem *et al.* [23]. The properties of the DES are strongly affected by the composition, temperature, pH value and water content [24]. Investigating DES properties under different conditions determines the suitability of the DES for use in organic synthesis applications [16], biotransformations [1,10], biomasss processing and electrochemistry [9].

This paper studies the physical properties of novel TBABr-based DESs, including density, viscosity and ionic conductivity. Measurements were made at different HBD percentages in the range of 66.7% to 90.0%, temperatures between 303 and 333 K and at atmospheric pressure, 1.05 atm. The following DESs were studied: combinations of TBABr with alcohol-based HBDs such as ethylene glycol, 1,3-propanediol, 1,5-pentanediol and glycerol. The physical properties of TBABr-based DES with combinations of these HBD have not been studied. The experiments studied the effects of HBD percentages, type of HBD and temperature changes on DESs, with a view to better understanding the properties of the DESs.

## 2. Results and Discussion

**Figure 1:**
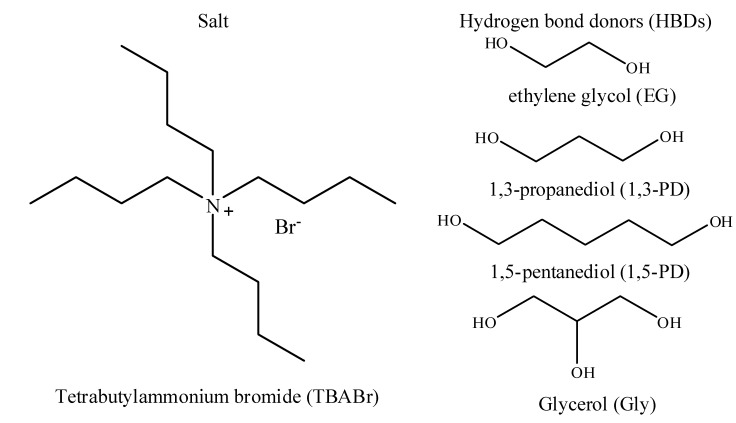
Structure of TBABr and HBDs used to synthesis the DESs.

Novel DESs based on TBABr salts with a few HBDs were successfully synthesized. The structures of TBABr salt and HBDs used in the syntheses of DESs include ethylene glycol (EG), 1,3-propanediol (1,3-PD), 1,5-pentanediol (1,5-PD) and glycerol (Gly) (Figure 1).

DESs in liquid phase at room temperature were labeled as TBABr:EG, TBABr:1,3-PD, TBABr:1,5-PD and TBABr:Gly. For each DES, five combinations consisting of 66.7%, 75.0%, 80.0%, 83.3% and 85.7% HBD were experimented on, except for the TBABr:Gly, which was studied in the range of 75.0% to 90.0% Gly. The percentage of HBD in DES are according to molar ratio. These DESs were liquids at room temperature. Below the stated percentage of HBD, the mixtures remained solid at room temperature. However, for TBABr:EG (66.7% EG) and TBABr:Gly (75.0% Gly), a slight precipitate was formed after three to four months, and heating is required prior to application to homogenize the DES.

### 2.1. Density

#### 2.1.1. The Effect of Temperatures on Density

**Figure 2:**
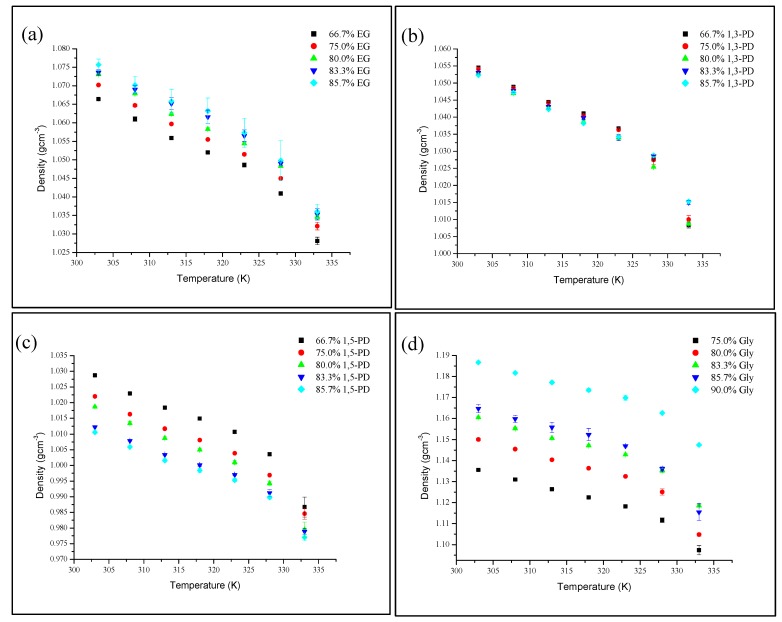
Density, *ρ* of DESs as a function of temperature K at different HBD percentages (**a**) TBABr:EG, (**b**) TBABr:1,3-PD, (**c**) TBABr:1,5-PD and (**d**) TBABr:Gly.

Density leads to an understanding of the liquid’s behavior. It is well known that density is drastically affected by the temperature and components of the liquid. It is important to know the effect of temperature on density in applications such as solvent design. The densities of DES at different temperatures are depicted in Figure 2. Three replicate readings were taken at each temperature for improved accuracy. There is no known literature on the densities of these DESs.

Density measurements were performed at temperatures ranging from 303 K to 333 K. TBABr:Gly exhibited the highest density, followed by TBABr:EG, TBABr:1,3-PD and TBABr:1,5-PD. All recorded densities of synthesized DESs were below 1.20 g·cm^−3^. The highest density was reported for TBABr:Gly, which reached a maximum of 1.1867 g·cm^−3^ of 90.0% Gly at 303 K. Meanwhile, TBABr:1,5-PD of 85.7% 1,5-PD showed the lowest density value of 0.9770 g·cm^−3^ at the highest tested temperature of 333 K.

The density of DESs decreases almost linearly with an increase in temperature (Figure 2). During heating, anions and cations in DESs vibrate. These vibrations can cause molecular rearrangements due to the weak interactions between ions, which in turn reduces the density of the liquid [25].

The results attained in this work were compared with the density of ChCl-based DES studied by Harris [14]. The density of TBABr:Gly at 90.0% Gly (1.1817 g·cm^−3^ at 308 K) was similar to the density of ChCl:Gly at 67% Gly (1.1810 g·cm^−3^ at 293 K). Meanwhile, TBABr:EG and TBABr:1,3-PD were found to have similar values to the DES of ChCl:1,4-butanediol at percentages of 75.0% to 95.0% 1,4-butanediol at 293 K (1.0210 to 1.0520 g·cm^−3^). Additionally, TBABr:1,5-PD had density values very similar to the DES formed from ChCl:1,2-butanediol at percentages of 80.0% to 95.0% 1,2-butanediol at 293 K (1.0090 to 1.0300 g·cm^−3^). It can be concluded that the density of TBABr-based DES is comparable to the density of other DESs, except for glucose-based DESs (1.2115 to 1.2978 g·cm^−3^ at 298.15 K to 358.15 K) [26]. The densities of TBABr-based DES are slightly lower than glucose-based DES.

ILs are generally denser than DESs, for example, the density of 1-alkyl-3-methyl-imidazolium iodide, [C_4_mim]I ranges from 1.3600 to 1.4340 g·cm^−3^ for the range of temperatures of 299.15 K to 388.15 K [27]. Additionally, the density of a few organic solvents were compared to the density of DESs. It was found that methanol has a density of 0.7822 g·cm^−3^, acetonitrile 0.7714 g·cm^−3^ and N,N-dimethylacetamide 0.9317 g·cm^−3^ at 303 K [28], all lower than the density of tested DESs.

#### 2.1.2. The Effect of HBDs on Density

**Figure 3:**
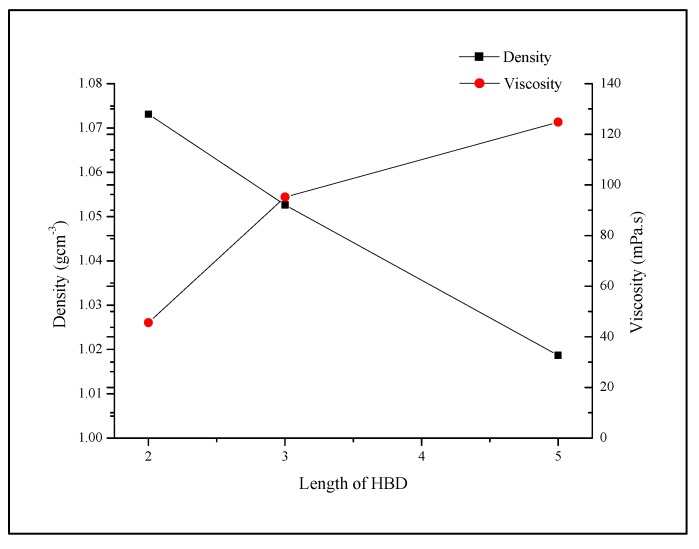
The graph of density, *ρ* and viscosity, η at 303 K *versus* the length of HBDs used in DESs.

Overall, at all tested temperatures as well as HBD percentages, TBABr paired with glycerol had the highest density when compared to the other synthesized DESs. It could be concluded that DES density can be highly affected by the type of the HBDs. The presence of three hydroxyl groups on HBD in TBABr:Gly was a contributing factor to the increase of density value as shown by the results. A 3D network of hydrogen bonds are formed through the interactions between glycerol and anions from TBABr [14]. The compact structure as a result of hydrogen bonds increases the density of DES. As Abbott *et al.* mentioned, the differences of density in DES are caused by the varying degrees of hydrogen bonds in these systems [29]. The density of DES with different carbon atom lengths were compared, where TBABr:EG, TBABr:1,3-PD and TBABr:1,5-PD each consist of two carbons, three carbons and five carbons chain, respectively. The density reduces drastically as the chain length of HBDs increases (Figure 3).

This result is supported by the interactions in DESs. A longer carbon chain of HBD in TBABr:1,5-PD decreases the hydrogen bond interactions due to steric hindrance. Consequently, the free volume tends to increase thus lowering the density of the liquid. The effect of a longer alkyl chain group on the density of an IL was previously studied by Ribe *et al.* [30]. A decrease in the density of R1-methylimidazolium tetrafluoroborate was observed when the alkyl chain length on the imidazolium cation increased.

#### 2.1.3. The Effect of HBD Percentages on Density

**Figure 4:**
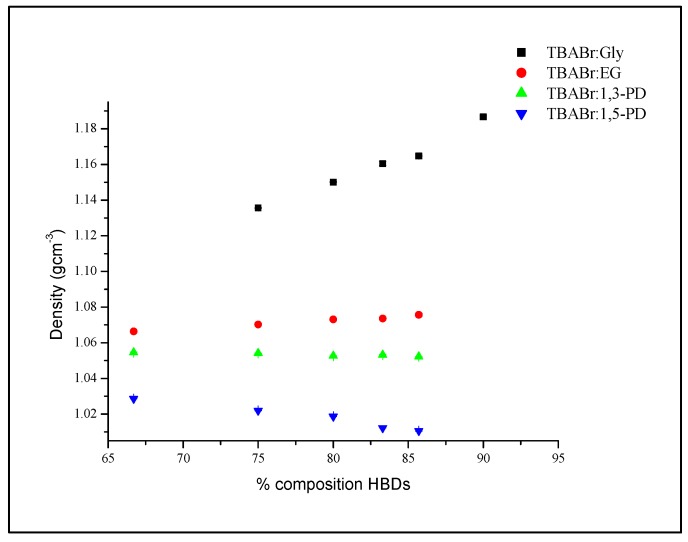
Plot of density, *ρ versus* HBD percentages at 303 K for alcohol HBDs based DES.

HBDs were added to the TBABr salt in amounts ranging from 66.7% to 85.7% for TBABr:EG, TBABr:1,3-PD and TBABr:1,5-PD. Meanwhile, 75.0% to 90.0% of Gly was added to TBABr:Gly. As can be seen in Figure 4, the effect of adding HBD into DES is very small, except in TBABr:Gly.

There was a remarkable increase in the density of TBABr:Gly from 1.1356 to 1.1867 g·cm^−3^ when Gly was added. Similarly, the density increased from 1.0664 to 1.0757 g·cm^−3^ as the EG was added to salt, which is barely observable from the graph. Meanwhile, the TBABr:1,5-PD showed an opposite trend, where the density decreased slightly from 1.0287 to 1.0106 g·cm^−3^ when 1,5-PD was added to TBABr salt. There was no visible change in the density of TBABr:1,3-PD when 1,3-PD was added since the value changed on slightly (by 0.002), from 1.0545 to 1.0523 g·cm^−3^.

Different effects of HBD percentages on density of DES system were observed. This might be due to the ways the HBD wraps around the Br anion, which is responsible for the size of the complex formed. Hence, it could affect the packing structure and density of DESs.

### 2.2. Viscosity

Viscosity is an internal friction measurement of a moving fluid which describes the resistance of a substance to flow [16]. In comparison to organic solvents, DESs have higher viscosity leading to some difficulties in handling, stirring and also filtering. The liquid viscosity is important in selecting an appropriate solvent. The viscosity is strongly influenced by the ability of the liquid to transport the mass within the liquid, which is immensely responsible for any changes in the chemical reactions. The high viscosity of the DES causes the limited mobility of species within the DES, which in turn causes a low conversion of products, especially in enzymatic reactions. The viscosity study was performed at different temperatures and HBD percentages.

#### 2.2.1. The Effect of Temperatures on Viscosity

**Figure 5:**
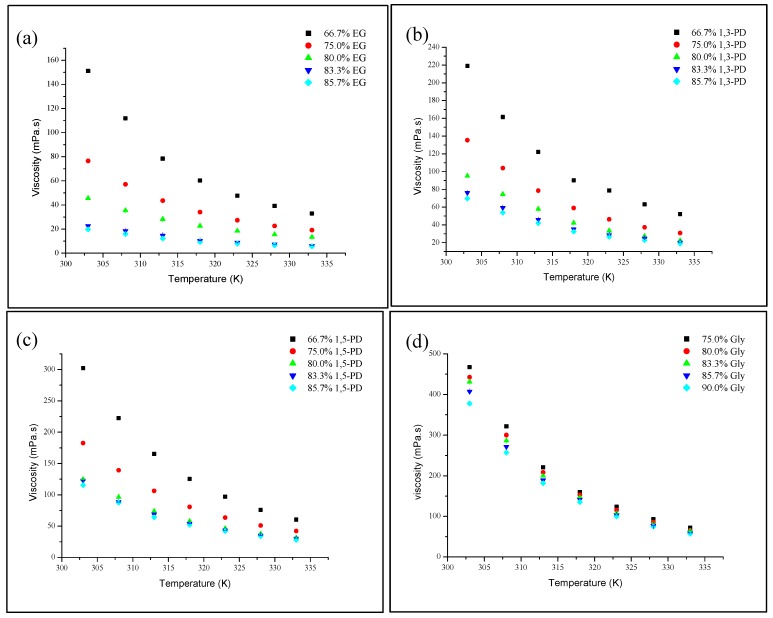
Viscosity, η of DESs as a function of temperature at different HBD percentages (**a**) TBABr:EG, (**b**) TBABr:1,3-PD (**c**) TBABr:1,5-PD and (**d**) TBABr:Gly.

The viscosity of DESs at different temperatures was studied in the same range as the density measurements (Figure 5).

It is noted that the highest viscosity at room temperature (303 K) belongs to TBABr:Gly (467.2 mPa·s). DESs viscosity reduces with an increase in temperature. Each DES attained its lowest viscosity at the highest tested temperature of 333 K, 5.7, 19.0, 28.0 and 57.5 mPa·s for TBABr:EG, TBABr:1,3-PD, TBABr:1,5-PD at 85.7% and TBABr:Gly at 90.0%, respectively. The vast difference in densities indicates that the DESs are highly sensitive towards temperature. Increased kinetic energy through heating may weaken the attractive forces between molecules [25]. The decrease in viscosity *vs*. temperature is a non-linear graph. Specifically, the viscosity decreases rapidly at low temperature and slowly approaches a similar value at higher temperature. These results are consistent with the reported literature [27,31].

#### 2.2.2. The Effect of HBDs Percentages on Viscosity

**Figure 6:**
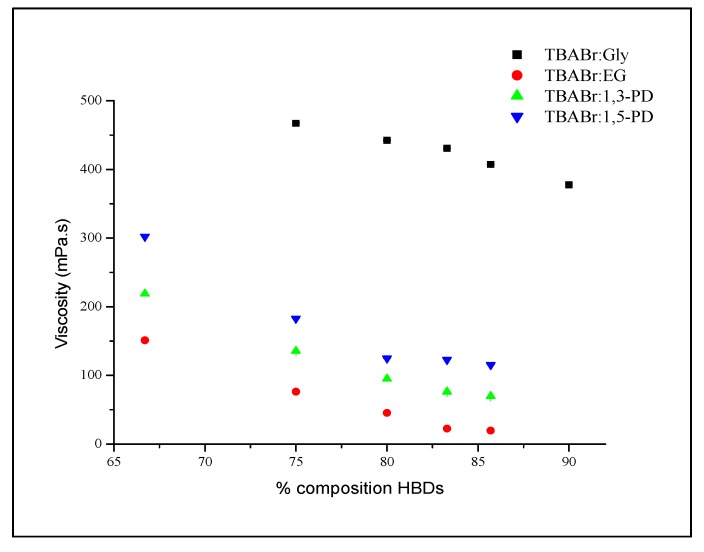
Plot of viscosity, η *versus* HBDs percentages at 303 K for alcohol HBD-based DES.

Meanwhile, the effect of HBD percentages on viscosity was investigated from 75.0% to 90.0% for TBABr:Gly and 66.7% to 85.7% for TBABr:EG, TBABr:1,3-PD and TBABr:1,5-PD. The addition of HBD decreased the viscosity of synthesized DESs (Figure 6). At 303 K, the viscosity of TBABr:Gly decreased slowly from 467.2 to 377.6 mPa·s upon addition of 75.0% up to 90.0% Gly into TBABr salt. This trend agrees with the results reported by Zhao *et al.* [17], who studied choline acetate and glycerol, but is in contrast with the work by Harris [14] and Abbott *et al.* [32], who studied choline chloride and glycerol in which the viscosity decreased only with the addition of ChCl salt. Their results were attributed to the ability of ChCl to disrupt the intermolecular hydrogen bonding of the 3-D glycerol structure, which led to a greater degree of freedom which decreases the viscosity of ChCl:glycerol. In contrast to their results, however there was no evidence of disruption of TBABr toward the glycerol. This could be mainly due to the type of salt affecting the viscosity of resulting mixtures.

Similarly, decrease of viscosities of TBABr:EG, TBABr:1,3-PD and TBABr:1,5-PD from 151.2 to 19.7 mPa·s, 219 to 69.8 mPa·s and 302.2 to 115.4 mPa·s respectively, were also noticed, when the HBD was added slowly from 66.7% to 85.7% at 303 K. The possible reason could be that the lower molar ratio of salt with HBD increases. The salt possibly acts as a bridge that connects the other ionic groups. A lower molar ratio of salt in DES caused decreased viscosity, due to the sparse network between different groups [33].

#### 2.2.3. The Effect of HBDs on Viscosity

As observed in density data, the TBABr-based DES has the highest viscosity when paired with the glycerol HBD. As TBABr:Gly contains more hydroxyl groups compared to the other DESs, it was expected to be the most viscous. The existence of extra hydroxyl groups creates more hydrogen bonds, which increases the attractive forces between molecules, making a liquid viscous. However, for the TBABr-based DESs with diol HBDs, the trends of viscosity are in contrast with the order of density data where TBABr:1,5-PD had the highest viscosity, followed by TBABr:1,3-PD and TBABr:EG.

As the number of carbon atoms increases, viscosity of DES increases while the density decreases. The structures of the HBDs are an important factor in determining density and viscosity. As an example, TBABr:1,5-PD with the longest alkyl chain among the tested HBDs in DESs had the highest viscosity and lowest density (Figure 3). The molecular chain of TBABr:1,5-PD became tangled due to its long carbon chain, which caused the increase in viscosity. As explained by Bonhote *et al.* [34], the high molar mass side chains in ILs have stronger van der Waals forces resulting in insufficient mobility and high viscosity. Hence, it can be concluded that when the length of HBD increases, viscosity increases but density decreases. The result is consistent with other research cited in the literature [27,30].

Abbott *et al.* [35,36] suggested the hole theory which describes the factors affecting the viscosity of ILs as well as DES. According to this theory, the ratio of ion radius to hole size within a liquid causes the difference in viscosity. If the hole is an equal or greater in size to its adjacent ion, the ion is able to move through the IL causing it to be less viscous [29]. Abbott *et al.* [36] mentioned that low viscosity can be obtained using a small quaternary ammonium cation such as the ethylammonium cation. The viscosity obtained at 313 K for ethylammonium cations paired with trifluoroacetamide_, _urea andacetamide are 256.0 mPa·s, 128.0 mPa·s and 64.0 mPa·s, respectively. Interestingly, the values are comparable to those seen with the much larger ions used in this study, tetrabutylammonium cations. However, there are other factors that may affect the ability of ions to move in DES other than the size of cations, such as the size of voids and radii of complex anions [36].

It is clear from this study that viscosities of DESs are higher when compared to most of the conventional solvents but almost similar to the ILs [4,10]. The viscosity of the synthesized DESs, ranging from 19.7 to 467.2 mPa·s at 303 K is higher than the viscosity of a few organic solvents such as methanol (0.6 mPa·s), ethanol (0.8 mPa·s), propanol (2.3 mPa·s), pyridine (1.5 mPa·s) at 293 K [37], benzene (0.5 mPa·s) and tetradecane (1.7 mPa·s) at 313 K and 6.8 atm [38]. The ILs have a wide range of viscosities. Ribe *et al.* [30] published the viscosity of ILs ranging from 10.7 to 284.1 mPa·s for BMIM[BF_4_] and 20.8 to 895.3 mPa for OMIM[BF_4_] at temperatures from 283 K to 363 K. Ochedzanet *et al.* [39] measured the viscosity for a variety of ILs at temperatures ranging from 293 K to 343 K, including C_4_MIM[AlCl_4_] at 8.7 to 34.0 mPa·s, C_6_MIM[AlCl_4_] at 15.3 to 68.0 mPa·s, C_6_-Py[AlCl_4_] at 19.0 to 65.3 mPa·s and C_4_-4-mpy[AlCl_4_] at 10.5 to 49.4 mPa·s.

### 2.3. Ionic Conductivity

#### 2.3.1. The Effect of Temperatures on Ionic Conductivity

**Figure 7:**
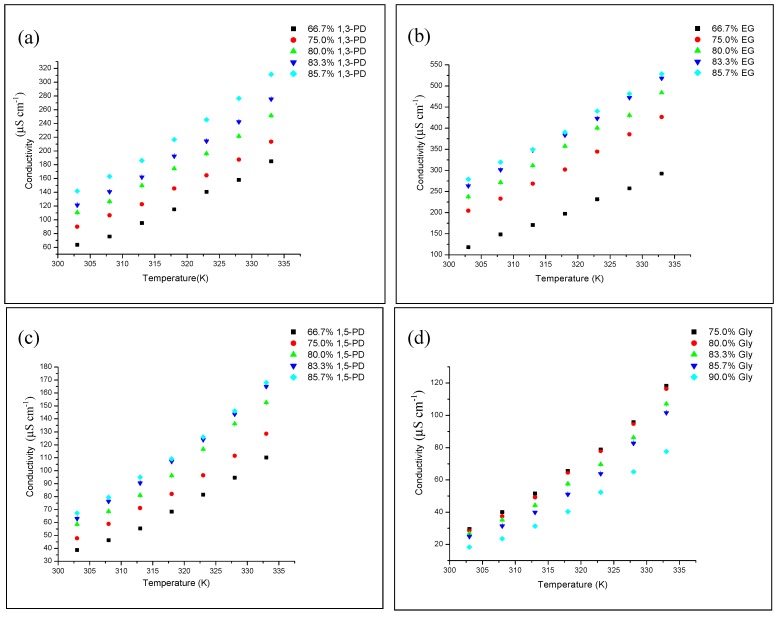
Ionic conductivity, *κ* of DESs as a function of temperature for different HBD percentages (**a**) TBABr:EG, (**b**) TBABr:1,3-PD (**c**) TBABr:1,5-PD and (**d**) TBABr:Gly.

Ionic conductivity was experimentally determined at different temperatures (Figure 7). For all four different type of DESs, ionic conductivity increases with temperature. Kinetic energy from heat increases the frequency of collision between molecules leading to weak intermolecular forces and increased ionic conductivity of DES [33].

#### 2.3.2. The Effect of HBD Percentages on Ionic Conductivity

**Figure 8:**
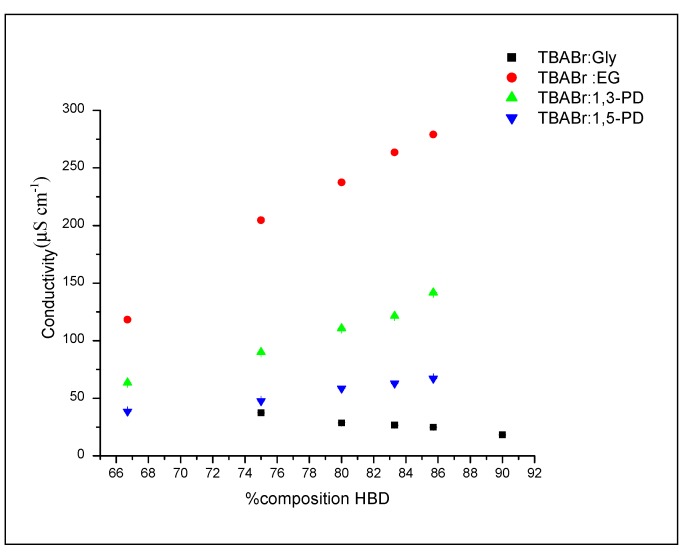
Plot of ionic conductivity, *κ versus* HBD percentages at 303 K for alcohol HBDs based DES.

Figure 8 illustrates the ionic conductivity of each DES as the amount of HBD added into DES mixture. The ionic conductivity of TBABr:EG, TBABr:1,3-PD and TBABr:1,5-PD increased from 66.7% to 85.7%, respectively, as the percentages of EG, 1,3-PD and 1,5-PD added increased. The increases of ionic conductivity arise from the high movement of charge carriers due to the decreases in viscosity. This can be explained by the Walden Rule, which describes the strong relationship between ionic conductivity and viscosity. The low viscosity of liquid results in high ionic conductivity [40,41].

Meanwhile, the ionic conductivity for TBABr:Gly decreased as percentages of glycerol in DES increased from 75.0% to 90.0% Gly. This occurred due to the decreased amount of TBABr salt in DES, resulting in lower ionic conductivity. From the viscosity results, it was proposed that ionic conductivity for TBABr:Gly would increase with the addition of HBD. However, it appeared to act differently, suggesting that there is a difference in the way glycerol complexes to the bromide ions of TBABr when compared to other systems. The Walden Rule has recently been used to describe IL ionic conductivity. However, deviation is possible as the rule applies to ions in infinite solution, in which interactions between ions are not considered. These results are also seen in other types of DES such as ChCl:1,4-butanediol and ChCl:1,2-butanediol [14].

As the comparisons were made, it can be concluded that TBABr:EG had the highest ionic conductivity (118.3 µS·cm^−1^ to 528.5 µS·cm^−1^), followed by TBABr:1,3-propanediol (63.7 µS·cm^−1^ to 311.5 µS·cm^−1^) and TBABr:1,5-pentanediol (38.7 µS·cm^−1^ to 168 µS·cm^−1^) when HBD was added from 66.7% to 85.7%. The lowest ionic conductivity was obtained for TBABr:glycerol valued 29.6 µS·cm^−1^ to 77.7 µS·cm^−1^ when HBD reduced from 90% to 75%.

The ionic conductivity of DES was lower than that of ILs. The value of ionic conductivity for pyridinium-based ILs such as [bpy][BF_4_] is 3,210 to 11,200 µS·cm^−1^, [bpy][CF_3_SO_3_] is 2,660 to 8,390 µS·cm^−1^ and [b_3_mpy][N(CN)_2_] is 10,270 to 25,800 µS·cm^−1^ in the 303 K to 333 K temperature range [41]. However, the ionic conductivity for a tetraethylammonium-based amino acid chiral IL such as [N2222][thr] (240 µS·cm^−1^ at 298 K) [40] was quite close to the ionic conductivity of TBABr:EG at 80.0% EG (237.5 µS·cm^−1^ at 303 K). Therefore, increasing the temperature or adding water to DES may increase ionic conductivity.

#### 2.3.3. The Effect of HBDs on Ionic Conductivity

The shorter alkyl chains of a diol HBD in TBABr:EG produce higher ionic conductivity due to the greater ion mobility of the salts in the mixture. Fewer van der Waals interactions make for the shorter carbon chains on HBD, which causes lower viscosity in DES mixtures [40]. Meanwhile, for the same HBD alkyl chain length, it was observed that the diol-based DES, TBABr:1,3-PD has higher ionic conductivity when compared to the triol-based DES, TBABr:Gly. Fewer hydroxyl groups in diol-based DESs produce fewer hydrogen bond networks than triol-based DESs, which lead to greater ion mobility and ionic conductivity. This was supported by the statement that the characteristics of the ionic structure, including size and shape determine its behavior in liquid, which strongly affects the ionic conductivity value [41].

## 3. Experimental Section

### 3.1. Materials

Tetrabutylammonium bromide (TBABr), 1,3-propanediol and 1,5-pentanediol were supplied by Fluka Chemical (Reidstr, Steinheim, Germany) with 98% purity. The hydrogen bond donor ethylene glycol was purchased from R&M (Essex, UK). Glycerol was purchased from J.T.Baker (Phillipsburg, NJ, USA). All chemicals were used without further purification.

### 3.2. Methods

#### 3.2.1. Synthesis of DESs

DESs mixtures were prepared accordingly to the method reported by Abbot *et al.* [12]. DESs were synthesized through mixing TBABr salt with different HBDs at various molar ratios. The mixtures were heated at 353 K for three to five hours at atmospheric pressure until a clear liquid was formed. However, some of DESs did not liquefy even after eight hours of mixing at temperatures as high as 373 K. The unsuccessful combinations were discarded from further investigation. Liquids DESs at room temperature were characterized for their density, viscosity and ionic conductivity (Table 1).

**Table 1:** Type of DES, percentage of HBD and abbreviations used for synthesis of TBABr based DES.

Type of DES	Percentage of HBD (%)	Abbreviations
TBABr:Ethylene glycol	66.7, 75.0, 80.0, 83.3, 85.7	(TBABr:EG)
TBABr:1,3-Propanediol	66.7, 75.0, 80.0, 83.3, 85.7	(TBABr:1,3-PD)
TBABr:1,5-Pentanediol	66.7, 75.0, 80.0, 83.3, 85.7	(TBABr:1,5-PD)
TBABr:Glycerol	75.0, 80.0, 83.3, 85.7, 90.0	(TBABr:Gly)

#### 3.2.2. Characterization of DESs

All synthesized DESs were initially dried in a vacuum drying oven set at 323 K for 24 h at 0.19 atm. They were stored in sealed laboratory vials and were kept in a desiccator over the silica gel. Studies for the density, viscosity and ionic conductivity of DESs were reported at temperatures ranging from 303 K to 333 K at percentages of 66.7% to 90.0% HBDs.

The viscosity of the DESs were measured using a Brookfield DV-II Pro viscometer (Middleboro, MA, USA) directly connected to Huber Compatible temperature controlled CC1 heating bath (Werner-von-Siemens-Strasse, Offenburg, Germany). The density of the DESs were measured using a Mettler Toledo Densito 30PX (Sonnenbergstrasse, Schwerzenbach, Switzerland) instrument. The ionic conductivity was measured using a conductivity meter from Mettler Toledo GmBH (model SevenEasy). The samples were heated in an oil bath, and the measurements were made after at least 20 min of warming in order to reach a good equilibrium of temperature between the DES sample and oil bath. All the measurements were repeated two to five times for each sample. The validity of the apparatus and the experimental methods used were tested by measuring the density, viscosity and ionic conductivity of distilled water under the same atmospheric pressure and temperatures as the DES sample.

## 4. Conclusions

DESs were successfully synthesized in various HBD percentages with the combination of four different HBDs, ethylene glycol, 1,3-propanediol, 1,5-pentanediol and glycerol. Experimental density, viscosity and ionic conductivity of the DESs from 303 to 333 K and at different HBD percentages were reported. The physical properties of different DESs are strongly influenced by the selection of HBD, temperature and percentage. The length of alkyl chain and numbers of hydroxyl groups on the HBD leads to changes in density, viscosity and ionic conductivity. The viscosity, density and ionic conductivity of synthesized DESs are quite similar to those of other DESs and ILs. It was evident that the density and viscosity of a DES decreases as temperature increases. However, the ionic conductivity of DESs increases with temperature. DESs made of longer HBD have higher viscosity, but lower density. The ionic conductivity of DES is always inversely related to the viscosity of the DES. The addition of HBD has a different effect in different type of DESs. The TBABr-based DESs had comparable density and viscosity to other DESs although they have a long tetrabutylammonium cation. The ionic conductivity of the prepared DESs is quite low. Increasing the temperature is the best solution to increase ionic conductivity. The results from this basic study of physical properties will be useful for the development of new DESs.
